# Using complex networks towards information retrieval and diagnostics in multidimensional imaging

**DOI:** 10.1038/srep17271

**Published:** 2015-12-02

**Authors:** Soumya Jyoti Banerjee, Mohammad Azharuddin, Debanjan Sen, Smruti Savale, Himadri Datta, Anjan Kr Dasgupta, Soumen Roy

**Affiliations:** 1 Bose Institute, 93/1 Acharya PC Roy Road, Kolkata 700 009, India; 2 Department of Biochemistry, University of Calcutta, 35 Ballygunge Circular Road, Kolkata 700 019, India; 3 Regional Institute of Ophthalmology, Calcutta Medical College and Hospital, Kolkata 700 073, India

## Abstract

We present a fresh and broad yet simple approach towards information retrieval in general and diagnostics in particular by applying the theory of complex networks on multidimensional, dynamic images. We demonstrate a successful use of our method with the time series generated from high content thermal imaging videos of patients suffering from the aqueous deficient dry eye (ADDE) disease. Remarkably, network analyses of thermal imaging time series of contact lens users and patients upon whom Laser-Assisted *in situ* Keratomileusis (Lasik) surgery has been conducted, exhibit pronounced similarity with results obtained from ADDE patients. We also propose a general framework for the transformation of multidimensional images to networks for futuristic biometry. Our approach is general and scalable to other fluctuation-based devices where network parameters derived from fluctuations, act as effective discriminators and diagnostic markers.

The field of Content Based Image Retrieval (CBIR) started with retrieval of specific images from a large array of images. Nowadays, CBIR is more generally referred to as Content Based Multimedia Information Retrieval (CBMIR) or simply MIR. Information retrieval in general can be conceived of as finding material of an unstructured nature that satisfies an information need from within large collections^1^. Applications of pictorial search into a database of images already existed in specialized fields like character recognition, face recognition, and, robotic guidance. IBM developed the first commercial CBIR system, called QBIC (Query by Image Content) in 1995^2^. At present, the basic problem is the creation of powerful content-based methods in order to enable or improve multimedia retrieval^3^. Many interesting and challenging scenarios arise in case of real time videos, e.g. surveillance, live cell imaging in life sciences, biomedical imaging etc.

Multidimensional imaging (MDI) is a ubiquitous and integral part of modern life. It is used in extremely diverse fields ranging from entertainment or surveillance on one hand to science, medicine or surgery on the other. To thrive like any other successful technology, a typical MDI technique needs to be inexpensive, portable, robust and high resolution to the extent possible. Additionally, successful coupling with efficient information retrieval algorithms could yield substantial premium. In this context, thermal imaging (TI) occupies an important place in MDI. TI is an economical and potent yet relatively unexplored technique in medical diagnostics. Lower imaging resolution and variability of steady state thermal behavior due to environmental thermal fluctuations are seen as primary reasons for the restrictive use of this powerful non-invasive method.

## Graph Theory in Computer Vision: A Toplogical Perspective

Many problems in image processing can be naturally mapped to energy minimisation approaches. However, such energy minimisation problems could be highly demanding from the computational point of view, as the general requirement is to minimise a non-convex function in a space with thousands of dimensions. Thankfully, dynamic programming can be used, but, only in a limited number of cases, where the energy functions have special forms^4^. In absence of such privileges, researchers typically used global optimisation techniques like simulated annealing^6^ or greedy algorithms^7^ for image smoothing which would be very slow for obvious reasons.

“Graph cut” approaches have come to be widely used in computer vision especially those that could be formulated in terms of energy minimisation. The essence of such approaches is that the basic technique is to construct a specialized graph on which the energy function to be minimized, such that the minimum cut on the graph in turn minimizes the energy. This follows from from the max-flow min-cut theorem that in a flow network, the amount of maximum flow is equal to capacity of the minimum cut. It was shown that maximising the flow through an image network is associated with the maximum *a posteriori* estimate of a binary image, introduction of sources and sinks make the problem efficiently solvable^5^. These approaches have been used successfully in a wide variety of vision problems including shape matching^8^, image restoration^9^,^10^, fingerprint recognition^11^, surface fitting^12^, stereo and motion^9^,^10^ and medical imaging^13^.

There also exists a body of work^14^,^15^ towards applying spectral encoding of a graph for indexing to large database of image features represented as Directed Acyclic Graphs (DAG). Databases of topological signatures can be indexed efficiently to retrieve model objects having similar topology. Significant research has been conducted on a general class of matching methods, called bipartite matching, to problems in object recognition The time complexity for finding such a matching in a weighted bipartite graph with *N* vertices was determined as  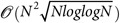
16.

Recent researches on image segmentation have used multi-resolution community detection methods in fluorescent lifetime microscopy^17^,^18^. Replica inference approaches have also been used towards unsupervised multiscale image segmentation^19^. Herein, we have used graph theory from a different perspective. Instead of object identification based on spatial correlations, we have exploited the relational topology of the image objects. This approach adds another angle to image segmentation and object identification, two classic problems in image processing.

## Time Series to Networks

A large number of approaches to analyze time series have been proposed over time. These range from time-frequency methods, such as Fourier and wavelet transforms^20^,^21^,^22^, to nonlinear methods, such as phase-space embeddings, Lyapunov exponents, correlation dimensions and entropies^23^,^24^,^25^. These techniques are helpful for summarizing the characteristics of a time series into compact metrics. Such brevity can be efficiently exploited to effectively understand the dynamics or to predict how the system will evolve over time. However, these measures preserve many but not all of the important properties of a given time series. Therefore, there is considerable research toward the identification of metrics that can capture the additional information or quantify time series in a completely new ways^26^,^27^,^28^,^29^.

Quite independent of the above, the field of complex networks has been extensively studied by itself and successfully applied in manifold instances in science, nature and engineering^30^,^31^. With significant advances being reported from various fields^32^,^33^,^34^,^35^,^36^,^37^,^38^,^39^,^40^,^41^,^42^,^43^, the importance of converting time series into networks is becoming increasingly clear over the last few years^44^.

## From Videos to Time Series and Thence to Networks

In this work, we furnish a new, simple and general route to information retrieval by combining developments from these disparate fields and show that such an approach can yield rich dividends for MDI in general and for diagnostics in particular. Indeed, following the broad framework proposed here, it is possible to construct inexpensive devices for non-invasive diagnostics and biometric based applications, which can perform successfully in real-time^45^. Our method consists of the following steps: (i) conversion of a given video or MDI into time series, (ii) conversion of the time series into a network, and finally (iii) analysing the network metrics to identify specific topological metric/(s) which can act as good discriminators for different videos.

## Advantages of the Present Approach

The process of conversion of any given video to a time series has been known for some time^46^. Albeit, to our knowledge, the fullest potential of this conversion in thermal imaging has not yet been exploited. Effective utilization of the vast research in time series analysis and related advances is obviously critical to gain liberal advantage of this transformation in information retrieval.

A network based representation of time series, provides us with an analytical tool that may allow object identification, which is not possible in many conventional image processing techniques. The uniqueness of the present identification approach is the use of analyses based on temporal instead of spatial distributions. As such network, based insights can be fed back for extraction of hidden image contents which are not evident from the spatial data alone.

Principal component analysis or PCA^47^ is a potent and widely used linear transform in signal and image processing, more specifically in image compression, blind signal separation, face and pattern recogntion^48^,^49^,^50^,^51^ etc. Essentially, PCA is a method for transforming a multidimensional dataset to a lower dimension. The basis vectors follow modes of the greatest variance, when the data is represented by PCA in the new coordinate system. However, PCA is also computationally expensive compared to many other processes like Fast Fourier Transformation. Herein, we show that in the present approach, the computationally expensive procedure of PCA adopted for dimensional reduction in conventional image processing can be safely circumvented. Obviously, dimension reduction achieved by our method would make feature identification of complex videos and images computationally far simpler. As detailed below, our approach effectively opens up the avenue of fluctuation based diagnostics in biomedical MDI. Hardware implementations of this method is extremely versatile, as it is smart, fast and portable^45^.

No established method, to our knowledge, has addressed the problem of the dynamics of thermal behavior from source thermal imaging data. Conventional image processing algorithms typically attempt appropriate segmentation, noise elimination and object identification like morphological changes or relative pixel dynamics. For videos, images extensive work has been done on motion tracking and this known to have important implications in contexts like security and surveillance.

Lastly, The present work of mapping biomedical videos into a time series and thence to a network should be implemented in diagnostic approaches, which need to record biomedical time-series data over a prolonged duration, perhaps without rest. The inconvenience or pain caused to a patient is imaginable.

## Applications to Diagnostics: Dry Eye Disease

While the approach proposed in this paper is very general, herein, we specifically concentrate on patients with Aqueous Deficient Dry Eye (ADDE) disease, contact lens users and patients who have undergone Lasik operations. We also investigate applications of our work in biometrics. ADDE is a disturbance in tear film physiology that leads to various abnormal states of ocular surface cells that elevate the incidence of ocular surface disorders and infection. ADDE represents one of the most common ocular pathologies and is a complex multifactorial disease characterized by an immune and inflammatory process that affects the lacrimal glands and ocular surface. Its diagnosis by assessment of the tear film has been extensively studied^52^,^53^,^54^. Most of the diagnostic approaches are based on either osmomolarity or evaporation of the tear film. Studies indicate that most ADDE measures do not capture the etiologies for dry eye, such as dysfunctional neurology, hormonal influences or the inflammatory nature of the condition. Another situation that may affect the alteration of the tear dynamics is the use of contact lens. Statistical studies of thermal fluctuation of healthy individuals (control) and ADDE patients where non-invasive TI was used, have been conducted recently^55^. Significant correlation of thermal fluctuations is found between left and right eye of control whereas this property is completely absent in eyes of ADDE patients. However, the problem of classification of dry eye either from collective or individual data remains unsolved. Also, parametric classification to differentiate or diagnose healthy and dry eye individuals is still unavailable. The mechanism proposed here shows that thermal fluctuation based approaches and a robust parametrization of such fluctuation by network mapping may be a powerful alternative approach to express the etiology of the eye. Throughout this paper, we use the terms dry eye and ADDE interchangeably. However, it should be especially noted that in medical literature, ADDE denotes only one of the spectra of alteration of ocular surfaces going by the name of the dry eye.

## Methods

### Ethics statement

All experiments analyzed herein were conducted after approval of the Ethics committee of Regional Institute of Opthalmology (RIO), Kolkata and were carried out in accordance with the approved guidelines of RIO. The research adhered to the tenets of the Declaration of Helsinki of the World Medical Association. Informed consent was obtained from all subjects.

**Figure 1:**
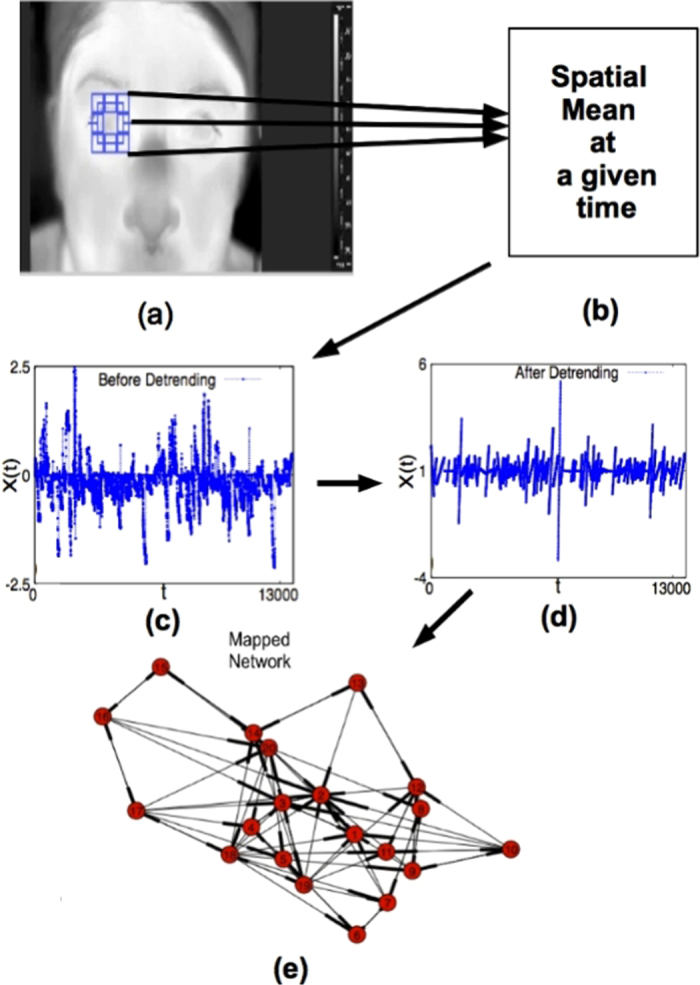
Outline of our method. (**a**) Thermal imaging of the right eye of a arbitrary individual, (**b**) spatial mean of the cropped region of interest at time *t*. Plot of pooled thermal time series data for both eyes of a particular group (like dry eye patients or healthy individuals) of all such thermal images (**c**) before detrending, and, (**d**) after detrending. Directed network obtained from mapping of detrended time series is shown in (**e**).

Figure 1 presents a schematic outline of our method. Specific details of our method are extensively discussed below.

### Thermal Imaging Setup

For our experiments, we used a Forward Looking Infra Red (FLIR) thermal camera, Model no. FLIR SC 305, FLIR Systems AB, Sweden. This camera is equipped with an RJ-45 gigabit Ethernet connection that supplies 16 bit 320 × 240 images at rates as high as 60 Hz along with linear temperature data. The video can be exported to several formats including AVI. In the FLIR SC 305 model, compression is used in the original video image and only the in built frame compression is used. Each frame is then cropped to select a region of interest (say eye, cheek etc). The camera was used with a thermal sensitivity of less than 0.05 °C at 30 °C, spatial, temporal and image resolution of 1.36 mrad, 9 frames per second and 320 × 240 pixels respectively, with spectral range between 7.5 and 13 mm.

### Details of Data Collection and Clinical Background of Subjects

Following are details of patient groups and healthy individuals for whom the thermal imaging videos were recorded for a duration of about 15 second and subsequently analysed:

- 36 Healthy individuals or for 72 eyes. Among them, 20 were female and 16 male, with a mean age of 28.4 years.
- 42 ADDE patients or for 84 eyes. Among them, 25 were female and 17 male, with a mean age of 35.2 years.
- 32 patients who had Lasik surgery or for 64 eyes. Among them, 18 were male and 14 female, with a mean age of 35.4 years.
- 29 Contact lens users or for 58 eyes. Among them, 15 were female and 14 male, with a mean age of 30.6 years.

For (d), videos were separately acquired for every individual when he or she was (i) wearing lens, and, (ii) not wearing lens.

The ocular surface temperature were recorded with eyes open and the subjects were asked not to blink during the recording. Noise of individual data could come from blinking of eyes if the videos are recorded for a longer duration. Probability of blinking of eyes tends to zero in a small duration like 15 second and therefore noise is negligible for the recorded data.

### Conversion of Videos to Time Series: PCA or a simple arithmetic mean?

**Figure 2:**
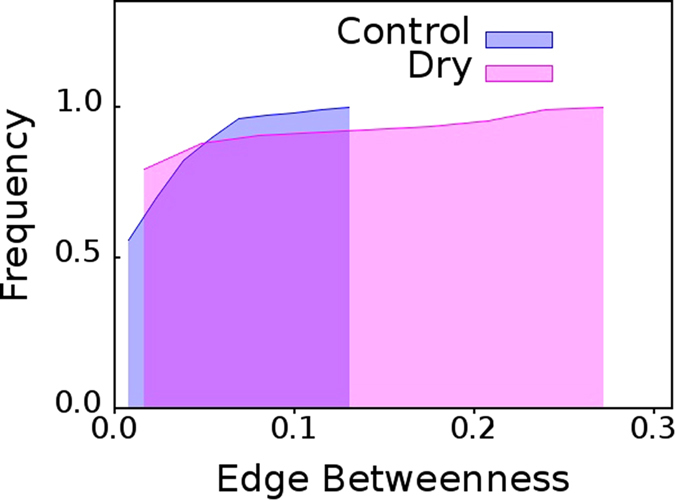
Healthy eye versus dry eye. Cumulative distribution of edge betweenness centrality for networks mapped from pooled thermal imaging time series for healthy eye and dry eye groups.

**Figure 3:**
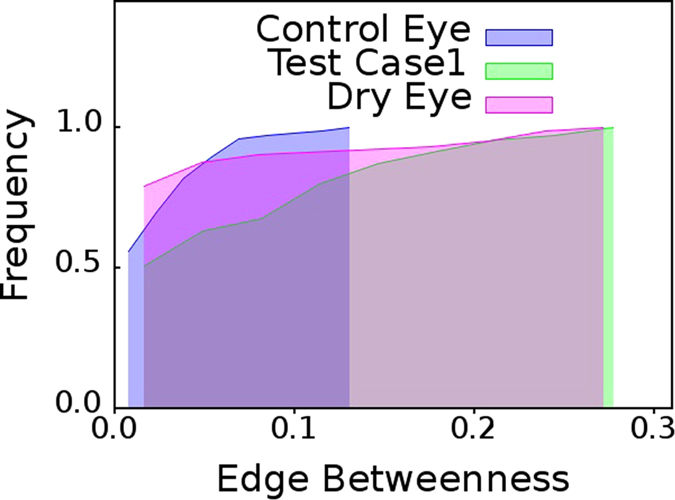
Test case: dry eye patient. Cumulative distribution of edge betweenness centrality, 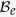, for networks mapped from pooled thermal imaging time series for individuals with healthy eyes (control) and dry eye patients shown in Fig. 2 and a test case. The test case is that of a dry eye patient whose cumulative 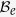 distribution is almost identical to the cumulative 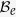 distribution of networks mapped from pooled thermal imaging time series data for all other dry eye patients.

**Figure 4:**
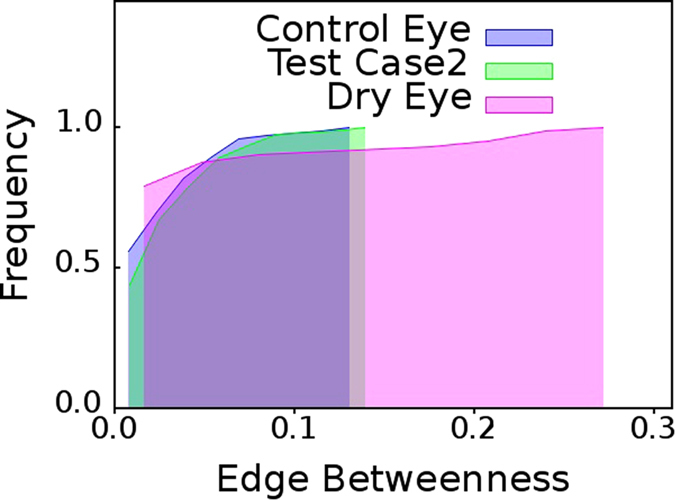
Test case: individual with healthy eye. Cumulative distribution of edge betweenness centrality, 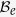, for networks mapped from pooled thermal imaging time series for individuals with healthy eyes (control) and dry eye patients shown in Fig. 2 and a test case. The test case is that of a healthy individual whose cumulative 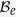 distribution is almost identical to the cumulative 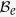 distribution of networks mapped from pooled thermal imaging time series data for all healthy individuals.

**Figure 5:**
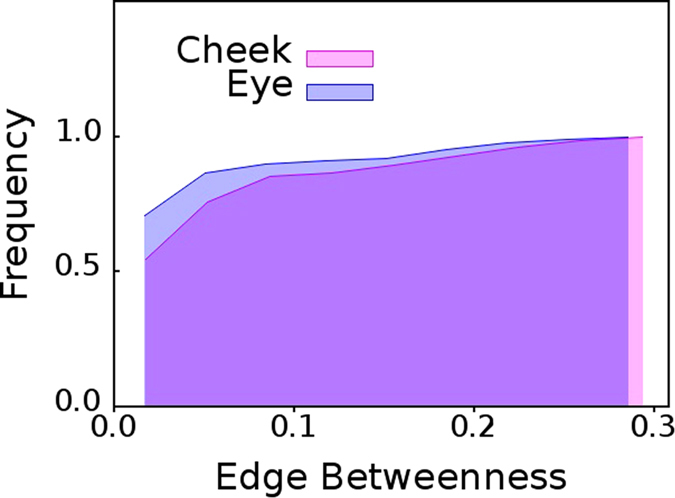
Cheek versus eyes of dry eye patients. Cumulative distributions of edge betweenness centrality, 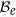 of networks mapped from pooled thermal imaging time series for 15 individuals with dry eyes, and, their cheeks.

**Figure 6:**
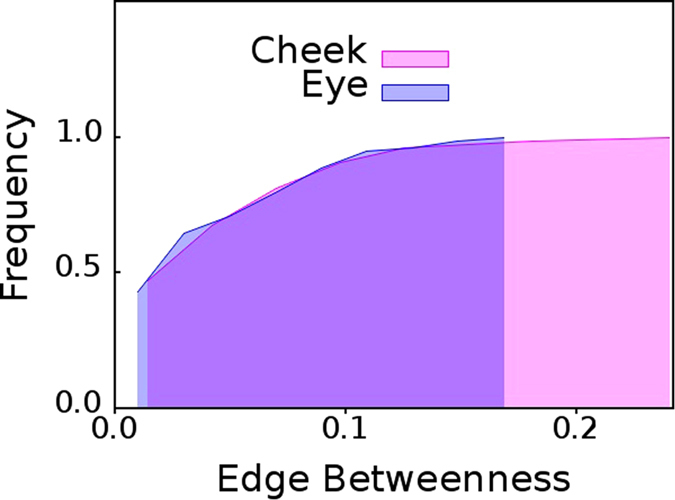
Cheek versus eyes of healthy individuals. Cumulative distributions of edge betweenness centrality, 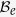 of networks mapped from pooled thermal imaging time series for 15 individuals with healthy eyes, and, their cheeks.

**Figure 7:**
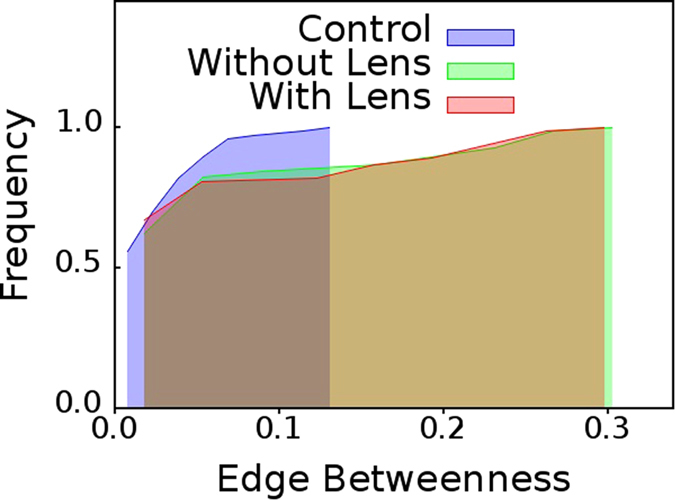
Contact lens users. Cumulative distributions of edge betweenness centrality, 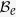 for networks mapped from pooled thermal imaging time series for individuals with healthy eyes, and, contact lens users. For the latter category, thermal imaging was conducted when they were: (**a**) wearing their contact lens, and, (**b**) not wearing their contact lens.

**Figure 8:**
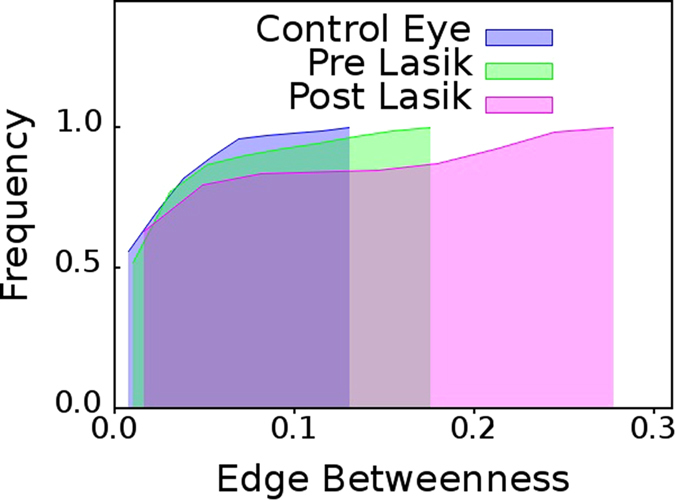
Patients who underwent Lasik surgery. Cumulative distributions of edge betweenness centrality, 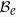 for networks mapped from pooled thermal imaging time series for individuals with healthy eyes and eyes of patients upon whom Lasik operation was conducted. Thermal imaging was conducted on the latter category, both, before they underwent surgery, and, after they underwent surgery.

Frame wise conversion of TI videos to time series has been implemented two ways: (1) Principal component analysis (PCA) of the cropped regions at each time instant, *t*, and, (2) Mean pixel value of the cropped region at each time instant, *t*. Thus, two entirely different time series of *N* points are obtained by the above methods. As is well-known, PCA is a frequently used technique for dimensional reduction in image processing^48^,^49^,^50^,^51^. As observed in Fig. S1 of supplementary information (SI), there is no significant variation of the principal components over time. The first three principal components collectively retain about 82% of variance of the data, in which the first principal component alone accounts for about 55%. On comparison between these two time series, we find that the classification of data, achieved by using mean pixel value as witnessed in Fig. 2, performs as good as the leading Principal Component, which is demonstrated in Fig. S2 of SI. The first three principal components capture about 82% of variance of the data, in which the first principal component alone accounts for over 55%. Naturally, we prefer the mean pixel value over PCA because, it: (i) is computationally far less inexpensive, (ii) is easy to implement in hardware and can be used to construct fast, smart and portable devices^45^, and, (iii) provides a statistically unambiguous and robust classification measure for a wide variety of circumstances. The latter is clearly evident in Figs 2, 3, 4, 5, 6, 7, 8. Thus we have taken the average pixel value as instantaneous temperature of the selected region, *τ*(*t*), at time instant, *t*. The initial instantaneous temperature, *τ*(0), at *t* = 0, is then set to 0. Thence, we obtain our final time series, 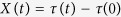.

### Detrending and Pooling Time Series

Some linear trend from decay of eye temperature is likely to be present in the time series generated from every video due to evaporation of water from the eye. In the event, that a large single time series is generated by pooling each of these smaller time series, a pseudo-periodicity could be created, which could mislead our predictions. So we detrend each time series separately to strip the accumulated time series from such a linear trend and normalize it by mean of all detrended values to fixed amplitude base line as shown in Fig. 1(c,d).

We independently pool the time series data of (1) both left and right eyes collectively, and, (2) the cheek of every individual in a given eye group (control or dry) and create a single time series. In all, we have eight classes of pooled time series. Throughout the rest of the paper, we often use 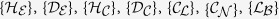 and 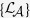 to denote: (i) healthy eyes (control group), (ii) dry eye group, (iii) cheek (control group), (iv) cheek (dry eye group), (v) group of contact lens users wearing lens, (vi) group of contact lens users not wearing lens, (vii) group of patients who underwent Lasik operations - before the procedure, and, (viii) group of patients who underwent Lasik operations - after the procedure. It should be noted that the individuals in (i) 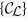 and 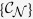, and, (ii) 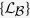 and 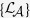 are identical.

### Creation of Networks from Time Series: Background

In literature, there are a few interesting approaches for time series to network mapping, based on different concepts such as correlations^56^,^57^, visibility^58^,^59^, recurrence analysis^60^, transition probabilities^61^,^62^,^63^ and phase-space reconstructions^64^,^65^. A complete list of all the proposed maps can be found in Donner *et al.*^66^ and references therein. These studies are able to capture that distinct properties of a time series can be mapped onto networks with distinct topological properties. These findings claim that it may be possible to differentiate different time series features using network metrics. But it was unclear as to how these network topological properties are related to the original time series. The main drawback of these maps 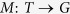 from the time series domain *T* to the network domain *G* however, is that they do not have a natural inverse mapping 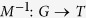. Recently, some researchers have tried to construct an inverse map^44^,^63^,^67^. Nonetheless, these methods are either sensitive to arbitrarily chosen parameters^44^,^67^ or are dependent on the information about the given map *M* for construction of an inverse mapping *M*^−1^ 63. Therefore, they are not immensely useful for real world networks, when *M* is not known in advance. Herein, we have used a recently developed time series to network mapping technique^68^. In our work, we have used only the forward mapping from time series to directed networks.

### Salient Features of Mapping Time Series to Directed Networks

We have followed methods well-established in literature^68^, for mapping the detrended time series of each of the eight groups mentioned above into eight distinct directed networks. These directed networks are denoted by 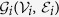, where, 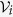 and 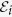 is the set of all nodes and edges respectively in network, and, 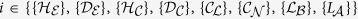. Further, for our case, we divide the Y-axis of Fig. 1(d) into 20 quantiles. It has been shown earlier^68^, that the time series to network mapping method used herein, is broadly independent of the number of equiprobable quantiles. Furthermore, the number of quantiles has no significant effect over the network topology^68^. We have chosen 20 quantiles to create a network with reasonable number of nodes and edges. We believe this to be an optimal number which can be accepted without the loss of generality, for reasons explained below. Creating too large a network will lead to unnecessary increase of computation time for calculating values of all node based and edge based network metrics, which are integral to our approach. Again, a very small number, e.g. 5 quantiles, is an unreliable choice for classification of groups using network properties. In our analyses, we have *ignored edge weights* because we are strongly interested *in capturing important thermal fluctuation transitions from one quantile to another rather than in how many times such transitions occurred.*

Nodes and edges of the networks mapped from the thermal videos have a clear physical interpretation. The nodes of the directed networks signifiy different ranges of temperature and edges represent the transitions from one temperature range to another. These obviously could be from a lower temperature regime to a higher temperature regime or vice versa. But, mere visual examination of networks derived by mapping from pooled thermal imaging time series is generally insufficient to classify between healthy eye and dry eye groups, irrespective of the network layout, as shown in Fig. S3 and Fig. S4 of Supplementary Information. Therefore, clearly identifying distinguishing properties of the thermal fluctuations for healthy individuals and diseased patients will be possible only upon thorough analyses of the directed networks using different topological network metrics and not merely by visual examination.

### Analyzing Directed Networks

All directed networks, thus obtained by mapping from thermal time series have been analyzed thoroughly using most known network metrics^38^, as detailed in Fig. S5 to Fig. S8 of Supplementary Information. As shown therein, among all network metrics, we find that edge betweenness centrality^36^,^42^, 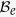, demonstrates significant discriminating power, a fact that can well be exploited to construct a functional, scalable device^45^. For directed networks, 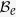, 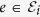 is the ratio of the number of directed shortest paths, 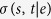, between node, *s*, and node, *t*, which pass through edge, *e*, and the total number of directed shortest paths, 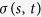, between node, *s*, and node, *t*, in the network. Mathematically, it is defined as:


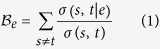


We then construct the *cumulative distribution* of 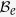, for each of the above groups.

## Results

### Cumulative Edge Betweenness Centrality Distribution for Dry Eye Versus Healthy Eye

From the cumulative 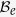 distributions shown in Fig. 2 it is clear that the networks mapped from pooled TI time series of eye for healthy eyes (control) group, 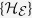, possess lower 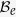 values where as the networks mapped from pooled TI time series of eye for dry eyes group, 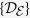, possess higher 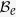 values. Since dry eye is a pathology or the eye; from Fig. 2 we get a hint that presence of edges with high 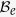 value could possibly be a suitable distinguishing, diagnostic feature for multiple pathological conditions in eyes. We subsequently find that this is indeed true to a large measure.

### Diagnosing Patients with Dry Eye

As aforementioned thermal imaging videos of 15 second duration were obtained for 42 dry eye patients, 36 healthy individuals, 32 individuals who underwent Lasik surgery, and, 29 regular contact lens users. For the Lasik Group, videos were obtained for all patients both before, and, after the surgery. For Contact lens users, videos were obtained both when they were: (a) wearing their lenses, and, (b) not wearing their lenses. In addition, TI videos of healthy individuals and patients with dry eye were further recorded for independent validation. These “test case” videos were of a total duration of 1 minute and obtained by pooling TI Videos of 15 second duration of the same individual. Such pooling is necessary, because: (i) blinking of eyes is not allowed in our experiment and it is difficult for most individuals not to blink even once in a minute, and, (ii) proper rest needs to be given to the eyes of all participants in the experiment.

From Figs 2, 3, 4, 5, 6, it is clear that the value of 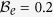 in the distribution of 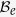, serves as a cut-off towards clearly distinguishing between healthy individuals and ADDE. If the cumulative 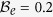 distribution of a subject, whose eyes have been thermally videographed for a minute, with adequate intermittent resting crosses 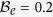, the subject is an ADDE patient. On the other hand, the cumulative 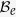 distribution of a healthy subject who has been similarly videographed, should clearly fall short of 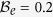. Thus, the present method has an inbuilt potential for incorporating a distance-like measure, 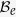, for clear image based segregation. It should be noted that the cutoffs seem statistically robust. Figures 5 and 6 have each been plotted for 15 pairs of randomly selected eyes from the original dataset of 42 pairs of dry eyes, and, 36 pairs of healthy eyes. The cutoffs remain unaltered from that derived in Fig. 2 for the original dataset.

### Adoption of a Double Blind Procedure

The following three completely disjoint groups have conducted this work: (*i*) opthalmologists *(Group A)*, (*ii*) experimentalists who undertook thermal imaging *(Group B)*, and, (*iii*) theoretical analysts, who had no exposure to the patients and were not present during videography *(Group C*). Thus, our diagnostic procedure is double blind and was conducted in the following steps.

- *Step 1: Group A* performed standard ADDE diagnostic tests upon classes of healthy individuals and patients but identified them only as belonging to either *Class 1* or *Class 2* to *Group B*; thus blanking out any clinical information. Additionally, two individuals of different clinical background, who were willing to be videographed for a longer duration were also supplied to *Group B*, again without any information about their clinical background.
- *Step 2: Group B* undertook non-invasive thermal imaging without any knowledge of the clinical background of their subjects and shared all videos of *Class 1* and *Class 2* and also of the two individuals to *Group C*.
- *Step 3: Group C* theoretically analysed the videos as detailed in this section and arrived at the cutoff of 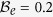. With help of this cutoff predicted by the training set data, namely, *Class 1* and *Class 2*; this group could predict whether the two test case individuals belonged to *Class 1* or to *Class 2*.
- *Step 4:* Lastly *Group A* confirmed that the inference of *Group C* is correct, i.e. Test case 1 is actually an ADDE patient, and, Test Case 2 is definitely a healthy individual, as predicted in Figs 3 and 4 respectively. Notably, these two test case eyes do not belong to any of the classes of the training sets, namely, *Class 1* and *Class 2*.

From the cumulative 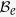 distributions shown in Fig. 3, we observe that cumulative 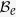 distribution of Test case 1 eye individual has larger shift toward right. The network mapped from TI time series for test case1 eye network has larger number of edges with high 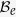 values which is quite similar to dry eye group. On the other hand, in the network obtained for the Test case 2 eye individual, the 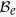 distribution is akin to that of the control eyes group as seen in Fig. 4. Therefore, we can reasonably infer that test case 1 should be a dry eye and test case 2 should be a healthy eye. Thus, in practice, diagnosis of dry eye is indeed possible by non-invasive thermal imaging of the eye of the patient for 60 second, by sufficiently resting the eyes of the arbitrary individual after every 15 second.

### Tagging Objects in Images

To identify possible biometric applications from our method, we also investigated the thermal fluctuations of other portions of the face apart from the eyes, namely, the cheek. The very same facial videos of both control eye and dry eye groups were studied for this purpose. This time however, the cheek portion was selected. 15 identical individuals were randomly selected from 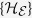 and 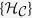. Similarly, 15 identical individuals were also randomly selected from 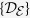 and 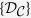. We *independently* pooled 15 detrended thermal times series, obtained from cheek and eye TI time series data for 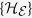, 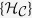, 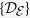 and 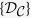.

Very interestingly, the cumulative edge betweenness, 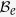, distributions for eye and cheek show completely different behavior. In the former case, the cumulative 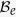 distribution of individuals with dry eyes selected from 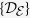 and 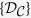 are almost identical, as can be observed from Fig. 5. However, in the latter case, cumulative 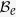 distribution of individuals with healthy eyes selected from 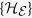 and 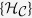 are very different, as visible in Fig. 6.

The above observations seem extremely important from the point of view of biometrics, for the following reasons. If there is any metabolic or functional object in which a biochemical or chemical activity is in progress, the temporal data transformed in network can provide a parametric identification of the status of the process, and the regional dependence of the same can further provide information regarding aberrations in functional status if any.

### Contact Lens Users

From the cumulative distributions shown in Fig. 7, it is clear that the contact lens users, (a) who are wearing lens, 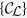, and, (b) who are not wearing lens, 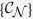; show characteristics similar to the dry eye group, 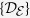, i.e., presence of edges with high 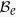 values when compared to the group of healthy individuals, 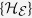. Contact lens users obviously also have unhealthy eyes due to myopia or hypermetropia. Significantly, removal of contact lens has almost no effect on the nature of the distribution.

### Patients Undergoing Lasik Surgery

From the cumulative 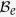 distributions shown in Fig. 8, we observe that cumulative 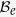 distribution of patients who have undergone Lasik surgery is also very similar to that of the cumulative 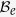 distribution of dry eye group; just like the case of contact lens users.

## Discussion

Edges of the directed networks represent the transitions from one temperature range to another; for example from a lower temperature regime to higher temperature regime or vice versa. For this reason, edge based network metrics would be very suitable for classification of thermal time series. Thus, it is not a surprise that edge betweenness centrality, 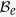, captures the most important transitions, representing significant thermal fluctuations, between two completely different temperature regimes, and, in the process exhibits such clear discriminating properties.

The results presented here illustrate that network based high content imaging can be a powerful tool for classification of objects in an image. The discriminatory potential of the said approach, which is in full display for ADDE patients in Fig. 2, contact lens users in Fig. 7, and, Lasik patients in Fig. 8; can hardly be derived using conventional video processing techniques.

As aforementioned, to our knowledge no established method has addressed the problem of the dynamics of thermal behavior from source thermal imaging data. In the present case, the image dynamics is a variation of another function, namely, temperature and the fluctuations are not due to environmental tuning alone as evident from above results. The function is actually a coupled manifestation of the thermal fluctuations of the environment and metabolic fluctuations. This coupling of fluctuations is difficult to understand using conventional techniques alone. The network metrics clearly imply that the coupling is different for healthy individuals and dry eye patients. In healthy individuals, the fluctuations in the eye remain independent of the other facial areas more exposed to the environment. In dry eye patients, this coupling is much stronger and the fluctuations of the eye and cheek are similar, implying that the metabolic control is lessened in the diseased state. Thus, dry eye subjects show significantly more fluctuations as compared to healthy individuals.

Our future plan is to design a method where parallel dynamics of different regions can be simultaneously evaluated. This will combine image processing algorithms like automatic object specific cropping and segmentation with topological properties of networks. While the immediate diagnostic potential of our findings for contact lens users or Lasik operated patients is unclear, the remarkable similarity of the cumulative 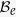 distribution for all eye diseases investigated here, namely, ADDE, contact lens users who obviously suffer from myopia or hypermetropia and Lasik operated patients is indeed striking. The last finding is specially interesting in context of the ongoing debate in ocular medicine as to whether Lasik operation actually leads to deterioration in the condition of the patients eye.

Last and certainly not the least, it may be noted that network based imaging approach can be exploited for designing efficient alert systems or biometric codes. Similarly, the technique can be applied to other forms of non-thermal high content imaging. The temporal dynamics provides us with a toolbox that would serve to compliment the knowledge gained from conventional image processing.

## Additional Information

**How to cite this article**: Banerjee, S. J. *et al.* Using complex networks towards information retrieval and diagnostics in multidimensional imaging. *Sci. Rep.* **5**, 17271; doi: 10.1038/srep17271 (2015).

## Supplementary Material

Supplementary Information

## References

[b1] Manning C. D., Raghavan P. & Schütze H. Introduction to Information Retrieval (Cambridge University Press, 2008).

[b2] Flickner M. *et al.* Query by image and video content: the QBIC system. IEEE Computer 28, 23–32 (1995).

[b3] Rowe L. A. & Jain R. ACM SIGMM report on future directions in multimedia research. ACM Trans. Multimedia Computing. Communications, and Application 1, 3–13 (2005).

[b4] Amini A., Weymouth T. & Jain R. Using dynamic programming for solving variational problems in vision. IEEE Transactions on Pattern Analysis and Machine Intelligence. 12, 855–867 (1990).

[b5] Greig D. M., Porteous B. T. & Seheult A. H. Exact maximum a posteriori estimation for binary images. J Royal Stat Soc, Series B. 51, 271–279 (1989).

[b6] Geman D. & Geman S. Stochastic relaxation, Gibbs distributions and the Bayesian restoration of images, IEEE Trans. Pattern Anal. Mach. Intell. 6, 721–741 (1984). 22499653 10.1109/tpami.1984.4767596

[b7] Besag J. E. On the statistical analysis of dirty pictures. J Royal Stat Soc, Series B. 48, 259–302 (1986).

[b8] Tang M., Gorelick. L., Veksler O. & Boykov. Y. GrabCut in One Cut. In Intl Conf. on Computer Vision (ICCV) Sydney, Austrailia (2013).

[b9] Boykov Y., Veksler O. & Zabih R. Markov Random Fields with efficient approximations. In IEEE Conf on Computer Vision and Pattern Recognition. pp 648–655 (1998).

[b10] Boykov Y., Veksler O. & Zabih R. Fast approximate energy minimization via graph cuts. IEEE Transactions on Pattern Analysis and Machine Intelligence. 23, 1222–1239 (2001).

[b11] Marcialis G. L., Roli F. & Serrau A. “Graph Based and Structural Methods for Fingerprint Classification”. Proceedings of the 7th intl conf on Multiple classifier systems, MCS ’07, pp 151–160, Springer-Verlag Berlin (2007).

[b12] Lempitsky V. & Boykov Y. Global Optimization for Shape Fitting. In IEEE Computer Vision and Pattern Recognition (CVPR) Minneapolis, USA (2007).

[b13] Kim J. *et al.* Incorporating spatial priors into an information theoretic approach for fMRI data analysis. In Medical Image Computing and Computer Assisted Intervention. pp 62–71 (2000).

[b14] Shokoufandeh A. & Dickinson S. Graph-Theoretical Methods in Computer Vision in Theoretical Aspects of Computer Science, Lecture Notes in Computer Science. 2292, Khosrovshahi G. B. *et al.* (Eds), pp. 148–174 (2002). Springer, Berlin Heidelberg (2002).

[b15] Kandel A., Bunke H., Last M. (Eds), Applied Graph Theory in Computer Vision and Pattern Recognition. Studies in Computational Intelligence. 52, Springer, Berlin Heidelberg (2007).

[b16] Gabow H., Goemans M. & Williamson D. An efficient approximate algorithm for survivable network design problems. Proc. of the Third MPS Conference on Integer Programming and Combinatorial Optimization 57–74 (1993).

[b17] Hu D., Sarder P., Ronhovde P., Achilefu S. & Nussinov Z. Community detection for fluorescent lifetime microscopy image segmentation. Proc. SPIE 8949, Three-Dimensional and Multidimensional Microscopy: Image Acquisition and Processing XXI (2014).

[b18] Hu D., Sarder P., Ronhovde P., Orthaus S., Achilefu S. & Nussinov Z. Automatic Segmentation of fluorescence lifetime microscopy images of cells using multi-resolution community detection. J. Microscopy. 253, 54–64 (2014). 10.1111/jmi.12097 PMC3893052 24251410

[b19] Hu D., Ronhovde P. & Nussinov Z. A replica inference approach to unsupervised multi-scale image segmentation. Phys. Rev. E 85, 016101 (2012). 10.1103/PhysRevE.85.016101 22400619

[b20] Korner T. W.. Fourier Analysis (Cambridge University Press, 1988).

[b21] Box G. E. P., Jenkins G. M. & Reinsel G. C. Time Series Analysis: Forecasting and Control (John Wiley & Sons, Inc., 2008).

[b22] Percival D. B. & Walden A. T. Wavelet Methods for Time Series Analysis (Cambridge University Press, 2006).

[b23] Strogatz S. H. Nonlinear Dynamics And Chaos: With Applications To Physics, Biology, Chemistry, And Engineering (Perseus Books Group, 1994).

[b24] Kantz H. & Schreiber T. Nonlinear Time Series Analysis (Cambridge University Press, 2003).

[b25] Campanharo A. S. L. O. *et al.* Searching chaos and coherent structures in the atmospheric turbulence above the Amazon forest. Phil Trans R Soc A 366, 579–589 (2008). 17698463 10.1098/rsta.2007.2118

[b26] Zhang J. & Luo X. & Small M. Detecting chaos in pseudoperiodic time series without embedding. Phys. Rev. E 73, 016216 (2006). 10.1103/PhysRevE.73.016216 16486267

[b27] Lai C., Chung P. & Tseng V. S. A novel two-level clustering method for time series data analysis. Expert Systems with Applications 37, 6319–6326 (2010).

[b28] Verplancke T. *et al.* A novel time series analysis approach for prediction of dialysis in critically ill patients using echo-state networks. BMC Medical Informatics and Decision Making 10, 1–7 (2010). 20092639 10.1186/1472-6947-10-4 PMC2828418

[b29] Ao S. Applied Time Series Analysis and Innovative Computing (Springer, 2010).

[b30] Albert R. & Barabási A. L. Statistical mechanics of complex networks. Rev. Mod. Phys. 74, 47 (2002).

[b31] Newman M. E. J. Networks: An Introduction (Oxford Univ. Press, UK, 2010).

[b32] Buldyrev S. V., Parshani R., Paul G., Stanley H. E. & Havlin S. Catastrophic cascade of failures in interdependent networks. Nature 464, 1025–1028 (2010). 20393559 10.1038/nature08932

[b33] Roy S. & Filkov V. Strong associations between microbe phenotypes and their network architecture. Phys. Rev. E 80, 040902(R) (2009). 10.1103/PhysRevE.80.040902 19905265

[b34] Dorogovtsev S. N., Goltsev A. V. & Mendes J. F. F. Critical phenomena in complex networks. Rev. Mod. Phys. 80, 1275 (2008).

[b35] Wuellner D. R., Roy S. & D’Souza R. M. Resilience and rewiring of the passenger airline networks in the United States. Phys. Rev. E 82, 056101 (2010). 10.1103/PhysRevE.82.056101 21230539

[b36] Newman M. E. J. & Girvan M. Finding and evaluating community structure in networks. Phys. Rev. E 69, 026113 (2004). 10.1103/PhysRevE.69.026113 14995526

[b37] Kaur Grewal R., Mitra D. & Roy S. Mapping networks of light-dark transition in LOV photoreceptors. 10.1093/bioinformatics/btv429, Bioinformatics (2015). 26209799

[b38] Filkov V. *et al.* Modeling and verifying a broad array of network properties. Euro. Phys. Lett. 86, 28003 (2009).

[b39] Kaur Grewal R. & Roy S. Modeling proteins as residue interaction networks. Protein & peptide letters, 22 923–933 (2015). 26216263 10.2174/0929866522666150728115552

[b40] Dandekar A. M. *et al.* Analysis of early host responses for asymptomatic disease detection and management of specialty crops. Crit. Revs. Immunol. 30, 277–289 (2010). 20370635 10.1615/critrevimmunol.v30.i3.50

[b41] Roy S. Systems biology beyond degree, hubs and scale-free networks: the case for multiple metrics in complex networks. Syst. Synth. Biol. 6, 31–34 (2012). 23730362 10.1007/s11693-012-9094-y PMC3424197

[b42] Girvan M. & Newman M. E. J. Community structure in social and biological networks. PNAS USA 99, 7821–7826 (2002). 12060727 10.1073/pnas.122653799 PMC122977

[b43] Banerjee S. J., Sinha S. & Roy S. Slow poisoning and destruction of networks: Edge proximity and its implications for biological and infrastructure networks. Phys. Rev. E 91, 022807 (2015). 10.1103/PhysRevE.91.022807 25768552

[b44] Strozzi F., Zaldívar J. M., Poljansek K., Bono F. & Gutiérrez E. From complex networks to time series analysis and viceversa: Application to metabolic networks. JRC Scientific and Technical Reports, EUR **23947**, JRC52892 (2009).

[b45] Roy S. *et al.* A system and method for analyzing videos of application or function for feature identification of the videos and related application or function. Indian Patent 628/KOL/2015 (2015).

[b46] Ishihara K., Kawagoe M. & Hasegawa R. Apparatus for and method of extracting time series image information. US Patent 5953439 (1999).

[b47] Jolliffe I. T. Principal Component Analysis, Second Edition, Springer-Verlag, New York (2002).

[b48] R. C. Gonzales R. C. & Woods R. E. Digital Image Processing. Prentice Hall, New Jersey, USA, Second edition (2002).

[b49] Barrett H. H. & Myers K. J. Foundations of Image Science. John Wiley & Sons, New Jersey, USA, Third edition (2013).

[b50] Petrou M. & Bosddogianni P. Image Processing: The Fundamentals. John Wiley & Sons, Inc., UK, Second edition (2000).

[b51] Turk M. A. & Pentland A. P. Face recognition using eigenfaces. IEEE Computer Soc. Conf. on Computer Vision and Pattern Recognition, CVPR ’91 10.1109/CVPR.1991.139758 (1991) June 3–6.

[b52] Tomilson A. & Khanal S. Assessment of Tear Film Dynamics: Quantification Approach. Clinical Science 3, 81–95 (2005). 10.1016/s1542-0124(12)70157-x 17131012

[b53] Khanal S., Tomlinson A., McFadyen A., Diaper C. & Ramaesh K. Dry Eye Diagnosis. Investigative Ophthalmology & Visual Science Cornea 49, 1407–1414 (2008). 10.1167/iovs.07-0635 18385057

[b54] Goins K. M. New Insights into the Diagnosis and Treatment of Neurotrophic Keratopathy. The Ocular Surface (Elsevier) 3, 96–110 (2005). 17131013 10.1016/s1542-0124(12)70158-1

[b55] Azharuddin M., Bera S. K., Datta H. & Dasgupta A. K. Thermal fluctuation based study of aqueous deficient dry eyes by non-invasive thermal imaging. Experimental Eye Research (Elsevier) 120, 97–102 (2014). 24457152 10.1016/j.exer.2014.01.007

[b56] Zhang J. & Small M. Complex network from pseudoperiodic time series: Topology versus dynamics. PRL 96, 238701 (2006). 10.1103/PhysRevLett.96.238701 16803415

[b57] Yang Y. & Yang H. J. Complex network-based time series analysis. Physica A 387 (2008).

[b58] Lacasa L., Luque B., Ballesteros F., Luque J. & Nuno J. C. From time series to complex networks: The visibility graph. PNAS USA 105, 4972–4975 (2008). 18362361 10.1073/pnas.0709247105 PMC2278201

[b59] Luque B., Lacasa L., Ballesteros F. & Luque J. Horizontal visibility graphs: Exact results for random time series. Phys. Rev. E 80, 046103 (2009). 10.1103/PhysRevE.80.046103 19905386

[b60] Marwan N., Donges J. F., Zou Y., Donner R. V. & Kurths J. Complex network approach for recurrence analysis of time series. Physics Letters A 373, 4246–4254 (2009).

[b61] Nicolis G., Cantu A. G. & Nicolis C. Dynamical aspects of interaction networks. Int. J. Bifurcation Chaos (World Scientific) 15, 3467 (2005).

[b62] Li P. & Wang B. H. Extracting hidden fluctuation patterns of Hang Seng stock index from network topologies. Physica A 378, 519–526 (2007).

[b63] Shirazi A. H. *et al.* Mapping stochastic processes onto complex networks. Journal of Statistical Mechanics: Theory and Experiment 07, P07046 (2009).

[b64] Xu X., Zhang J. & Small M. Superfamily phenomena and motifs of networks induced from time series. PNAS USA 105, 19601–19605 (2008). 19064916 10.1073/pnas.0806082105 PMC2604928

[b65] Gao Z. & Jin N. Complex network from time series based on phase space reconstruction. Chaos 19, 033137 (2009). 19792017 10.1063/1.3227736

[b66] Donner R. V. *et al.* Recurrence- based time series analysis by means of complex network methods. Intl J Bifurcation and Chaos 21, 1019–1046 (2011).

[b67] aguchi Y., Shimada Y., Ikeguchi T. & Aihara K. Transformation from complex networks to time series using classical multidimensional scaling. (In: ICANN ’ 09: Proceedings of the 19th International Conference on Artificial Neural Networks Heidelberg, Berlin: Springer-Verlag, 2009).

[b68] Campanharo A. S. L. O., Sirer M. I., Malmgren R. D., Ramos F. M. & Amaral L. A. N. Duality between Time Series and Network. PLoS ONE 6, e23378 (2011). 21858093 10.1371/journal.pone.0023378 PMC3154932

