# Supplementary material for: Using complex networks towards information retrieval and diagnostics in multidimensional imaging
Source: Sci Rep. 2015 Dec 2;5:17271. doi: 10.1038/srep17271 (PMC4667282; doi:10.1038/srep17271)
Supplement: Supplementary Information [file srep17271-s1.pdf]

SUPPLEMENTARY INFORMATION

Using complex networks towards information retrieval and diagnostics in multidimensional imaging

Soumya Jyoti Banerjee[1], Mohammad Azharuddin[2], Debanjan Sen[3], Smruti Savale[3], Himadri Datta[3], Anjan Kr Dasgupta[2] and Soumen Roy[1,*]

[1]Bose Institute, 93/1 Acharya PC Roy Road, Kolkata 700 009 India
[2]Department of Biochemistry, University of Calcutta 35 Ballygunge Circular Road, Kolkata 700 019, India
[3]Regional Institute of Ophthalmology, Calcutta Medical College and Hospital, Kolkata 700 073, India
*soumen@jcbose.ac.in

1 Mean value of pixels versus Principal Component Analysis

We have conducted a principal component analysis (PCA) of the cropped portion of the images obtained by videographing the eyes for the purpose of dimensional reduction. Let $Z_{ij}(t)$ denote the pixel image matrix at time t, of the cropped portion. In the main text, we chose $\langle Z(t) \rangle$, the mean value of pixels of the cropped region for each frame obtained by decomposition from the video. To contrast with $\langle Z(t) \rangle$, herein we study the time series of $\lambda_k(t)$, where, k denotes all principal components which are obtained from the matrix $Z_{ij}(t)$ at time, t.

We ensure that there is no significant variation of the principal components over time, as observed in Fig. S 1. The first principal component, $PC1$, alone retains about 55% of the total variance of the data and the first three principal components collectively retain about 82%. To contrast with the mean pixel value, $\langle Z(t) \rangle$; herein we choose to create a network from the time series of the first principal component, or the leading eigen value, $\lambda_1(t)$, obtained from the matrix $Z_{ij}(t)$ at time t. The procedure of creating the network from the time series of $PC1$ is exactly identical to that of creating the network from $\langle Z(t) \rangle$, as detailed in the main text.

Comparing Fig. 2 of main text and Fig. S 2 of SI, it is observable that there is *essentially no difference in the nature of classification* inferred from the cumulative distributions of edge betweenness centrality. As evident, the former has been obtained by using $\langle Z(t) \rangle$ and the latter by using the first principal component. Thus, calculating the spatial mean of pixels of the cropped portion essentially gives the same results and is obviously far less computationally expensive than PCA.

As evident from Figs. S 3 and S 4, it is evident that the networks cannot be differentiated upon mere visualisation. Further topological analyses are necessary.

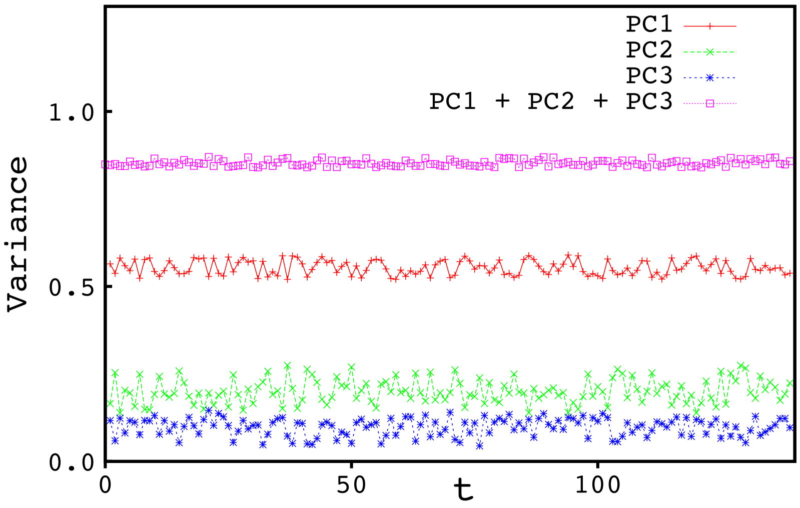

Supplementary Figure S 1: Fraction of the total variance captured individually and collectively by the first three principal components as a function of time.

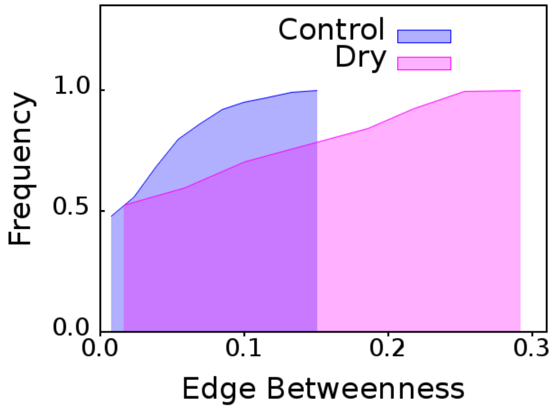

Supplementary Figure S 2: Cumulative distributions of edge betweenness centrality metric for networks mapped from pooled thermal imaging time series for healthy eye and dry eye groups using the first principal component obtained from the cropped portion of images.

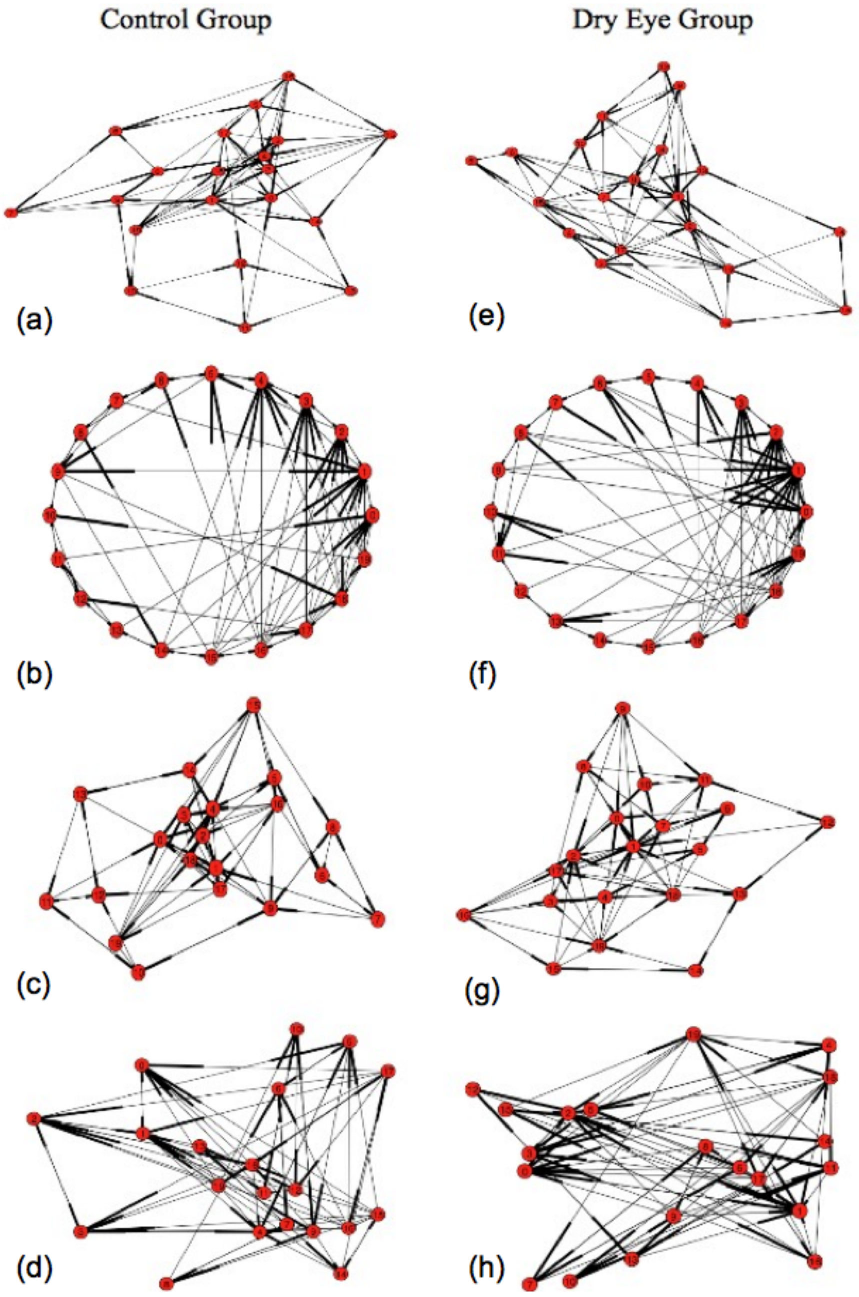

Supplementary Figure S 3: **Visualising networks using the mean value of pixels.** A network for healthy individuals and a network for dry eye patients, obtained after mapping from the pooled thermal imaging time series using the mean pixel value as detailed in text, is drawn in different layouts. (a),(b),(c),(d) depict the networks for healthy individuals. (e),(f),(g),(h) represent the networks of ADDE patients. (a) & (e) represent spring layout, (b) & (f) represent circular layout, (c) & (g) represent Fruchterman-Reingold layout, and, (d) & (h) represent random layout. Obviously, the networks cannot be differentiated by mere visualisation. Further topological analyses are necessary.

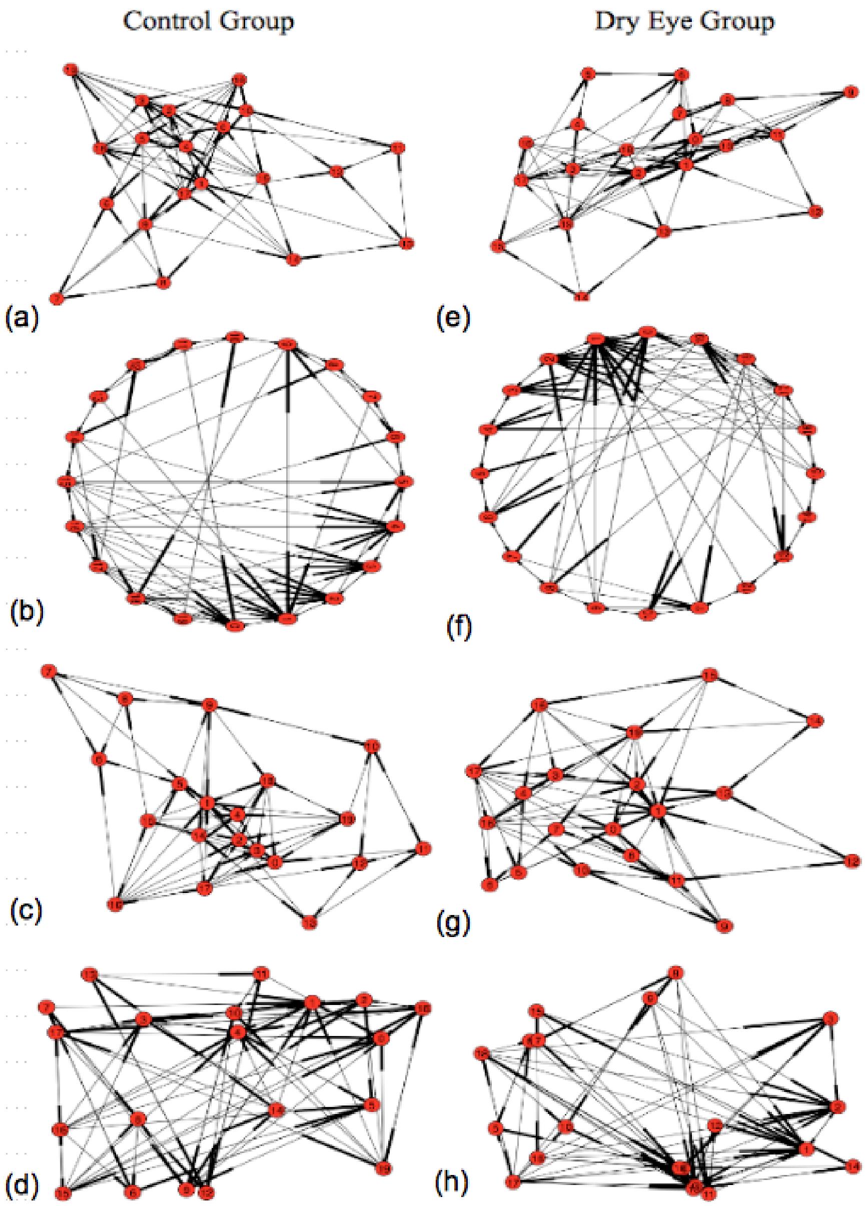

Supplementary Figure S 4: **Visualising networks from the leading Principal Component.** A network for healthy individuals and a network for dry eye patients, obtained after mapping from the pooled thermal imaging time series using the leading principal component as detailed in text, is drawn in different layouts. (a),(b),(c),(d) depict the networks for healthy individuals. (e),(f),(g),(h) represent the networks of ADDE patients. (a) & (e) represent spring layout, (b) & (f) represent circular layout, (c) & (g) represent Fruchterman-Reingold layout, and, (d) & (h) represent random layout. Obviously, the networks cannot be differentiated by mere visualisation. Further topological analyses are necessary.

2 Analyses of networks using multiple metrics

Distributions of topological metrics have been studied for both $PC1$ and $\langle Z(t) \rangle$ and observed to exhibit similar behaviour. To avoid redundancy, here we present results for only $\langle Z(t) \rangle$ in Figs. S 5, S 6, S 7 and S 8. Below, we examine standard network metrics like degree, closeness centrality and betweenness centrality of nodes. Similarly, for edges, we study edge proximity and edge betweenness centrality of edges. Edge betweenness centrality clearly serves as the best classifier between healthy individuals and patients with dry eye disease. This is true for both $PC1$ and $\langle Z(t) \rangle$ as reflected in Fig. S 2 and Fig. 2 of main text respectively.

2.1 Degree of nodes

The degree of a node is the total number of edges incident on that node. Since our networks are directed, degree of each node is the sum of its in-degree and out-degree. Cumulative degree distribution for control and ADDE group is shown in Fig. S 5.

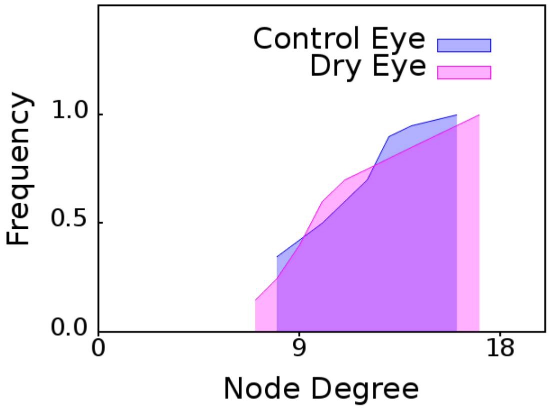

Supplementary Figure S 5: Cumulative degree distribution for networks mapped from pooled thermal imaging time series for healthy eye and dry eye groups.

2.2 Closeness of nodes

Closeness centrality, C_i, of any node, i, is the inverse of the sum of its shortest distance $d(i, j)$, calculated using the directed shortest paths, with every other node, j, in the network. Thus,

$$C_i = \frac{N-1}{\sum_j d(i,j)} \tag{1}$$

where N is the total number nodes in the network. Cumulative distributions of C_i for control and ADDE group is plotted in Fig. S 6.

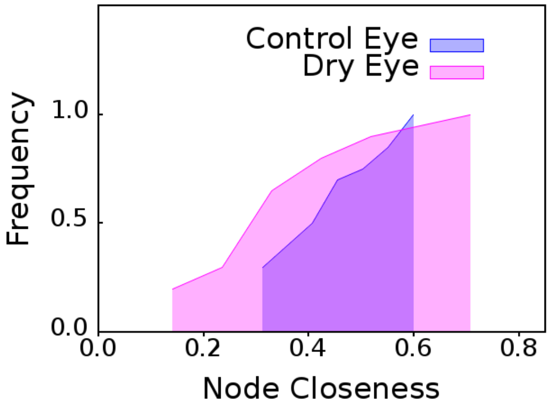

Supplementary Figure S 6: Cumulative distributions of node closeness, C_i, for networks mapped from pooled thermal imaging time series for healthy eye and dry eye groups.

2.3 Betweenness of nodes

Betweenness centrality, B_i, of a node, i, is the ratio of the number of directed shortest paths, $\sigma(s,t|i)$, between node, s, and node, t, which pass through node, i, and the total number of directed shortest paths, $\sigma(s,t)$, between node, s, and node, t, in the network. Mathematically, it is defined as:

$$B_i = \sum_{s \neq t} \frac{\sigma(s,t|i)}{\sigma(s,t)} \tag{2}$$

Cumulative distribution of B_i for control and ADDE group is demonstrated in Fig. S 7.

2.4 Edge proximity of edges

Edge proximity of any edge, e, is the inverse of the sum of it's shortest distance $d(e,f)$, with every other edge, f, in the network. Thus,

$$E_P = \frac{M-1}{\sum_f d(e,f)}, \tag{3}$$

where, M is the total number edges in the network. Cumulative distributions of edge proximity for control and ADDE group is shown in Fig. S 8.

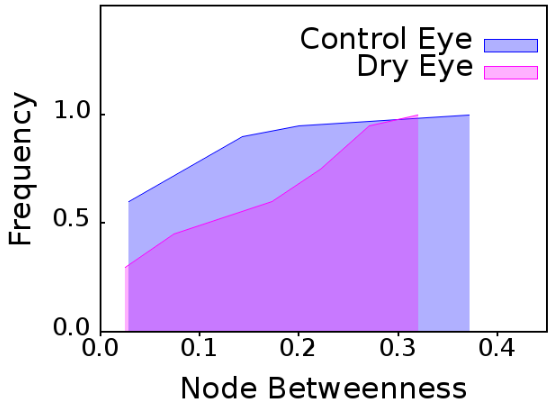

Supplementary Figure S 7: Cumulative distributions of node betweenness, B_i, for networks mapped from pooled thermal imaging time series for healthy eye and dry eye groups.

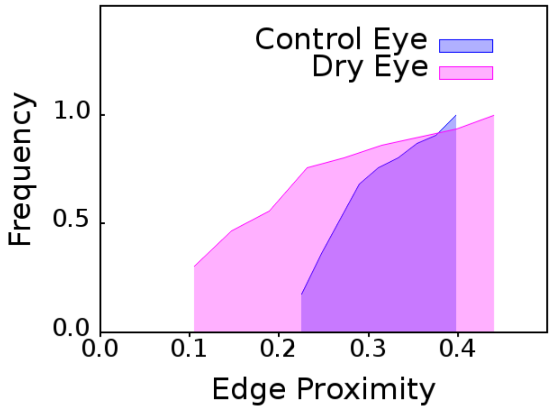

Supplementary Figure S 8: Cumulative distributions of edge proximity centrality for networks mapped from pooled thermal imaging time series for healthy eye and dry eye groups.
